# Lysophosphatidic Acid Receptor Signaling in the Human Breast Cancer Tumor Microenvironment Elicits Receptor-Dependent Effects on Tumor Progression

**DOI:** 10.3390/ijms24129812

**Published:** 2023-06-06

**Authors:** Matthew G. K. Benesch, Rongrong Wu, Xiaoyun Tang, David N. Brindley, Takashi Ishikawa, Kazuaki Takabe

**Affiliations:** 1 Department of Surgical Oncology, Roswell Park Comprehensive Cancer Center, Buffalo, NY 14263, USA; matthew.benesch@roswellpark.org; 2 Department of Breast Surgery and Oncology, Tokyo Medical University, Tokyo 160-8402, Japan; rongrong.wu@roswellpark.org (R.W.); tishik55@gmail.com (T.I.); 3 Cancer Research Institute of Northern Alberta, Department of Biochemistry, University of Alberta, Edmonton, AB T6G 2H7, Canada; xtang2@ualberta.ca (X.T.); david.brindley@ualberta.ca (D.N.B.); 4 Department of Gastroenterological Surgery, Yokohama City University Graduate School of Medicine, Yokohama 236-0004, Japan; 5 Division of Digestive and General Surgery, Niigata University Graduate School of Medical and Dental Sciences, Niigata 951-8520, Japan; 6 Department of Breast Surgery, Fukushima Medical University School of Medicine, Fukushima 960-1295, Japan; 7 Department of Surgery, University at Buffalo Jacobs School of Medicine and Biomedical Sciences, State University of New York, Buffalo, NY 14263, USA

**Keywords:** autotaxin, bioinformatics, lysophosphatidate, novel therapeutics, signal transduction, tumor progression

## Abstract

Lysophosphatidic acid receptors (LPARs) are six G-protein-coupled receptors that mediate LPA signaling to promote tumorigenesis and therapy resistance in many cancer subtypes, including breast cancer. Individual-receptor-targeted monotherapies are under investigation, but receptor agonism or antagonism effects within the tumor microenvironment following treatment are minimally understood. In this study, we used three large, independent breast cancer patient cohorts (TCGA, METABRIC, and GSE96058) and single-cell RNA-sequencing data to show that increased tumor *LPAR1*, *LPAR4*, and *LPAR6* expression correlated with a less aggressive phenotype, while high *LPAR2* expression was particularly associated with increased tumor grade and mutational burden and decreased survival. Through gene set enrichment analysis, it was determined that cell cycling pathways were enriched in tumors with low *LPAR1*, *LPAR4*, and *LPAR6* expression and high *LPAR2* expression. LPAR levels were lower in tumors over normal breast tissue for *LPAR1*, *LPAR3*, *LPAR4*, and *LPAR6*, while the opposite was observed for *LPAR2* and *LPAR5*. *LPAR1* and *LPAR4* were highest in cancer-associated fibroblasts, while *LPAR6* was highest in endothelial cells, and *LPAR2* was highest in cancer epithelial cells. Tumors high in *LPAR5* and *LPAR6* had the highest cytolytic activity scores, indicating decreased immune system evasion. Overall, our findings suggest that potential compensatory signaling via competing receptors must be considered in LPAR inhibitor therapy.

## 1. Introduction

As the most common cancer in women, accounting for about 25% of all cancer diagnoses with a lifetime risk of one in eight [1], breast cancer remains a challenging entity despite modern treatment modalities. When treated at the localized disease stage, 5-year survival rates approach 99% percent [2]. However, nearly 30% of breast cancer cases are diagnosed with lymph node involvement and 6% with metastasis, thereby lowering 5-year survival rates to 86% and 30%, respectively [2]. In the United States alone, about 43,000 patients continue to die annually from breast cancer, primarily due to relapsed disease that has become resistant to treatment [3,4]. Decoding and therapeutically targeting mechanisms of treatment resistance and failure are the primary areas of modern breast cancer research that will most likely have the greatest capacity to further improve survival trends [5].

**Figure 1:**
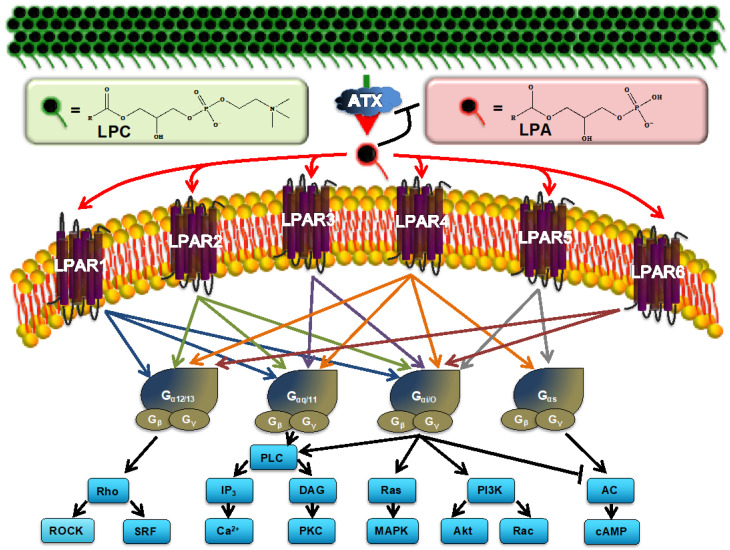
Overview of the current model of lysophosphatidic acid receptor (LPAR) signaling in breast cancer. Extracellular autotaxin (ATX) produces bioactive LPA from essentially biologically inert lysophosphatidylcholine (LPC), which is present in human plasma at 200 μM, via hydrolysis of the choline headgroup. LPA levels are normally present at 0.1–1 μM. This ratio is depicted by the green (LPC) and red (LPA) icons. LPA signals through at least six G-protein-coupled receptors to elicit a wide range of cellular responses. Signaling through these receptors may be redundant and/or antagonistic depending on the heterotrimeric G-protein that is coupled to the LPAR. G_α_/_β_/_γ_, G-protein alpha/beta/gamma subunits; Rho, Rho GTPase; ROCK, Rho-associated protein kinase; SRF, serum response factor; IP3, inositol triphosphate; PLC, phospholipase C; DAG, diacylglycerol; PKC, protein kinase C; MAPK, mitogen-activated protein kinase; PI3K, phsophoinositide 3-kinase; Akt, protein kinase B; AC, adenylate cyclase; cAMP, cyclic adenosine monophosphate.

Lysophosphatidate, also known as lysophosphatidic acid (LPA), is a potent bioactive signaling molecule that orchestrates many physiological pathways involved in embryogenesis and wound healing [6,7,8]. It is primarily produced in the extracellular space by the lysophospholipase D activity of autotaxin (ATX) via the hydrolysis of the choline head group [9] (Figure 1). LPA signals through six G-protein-coupled receptors (LPARs) to mediate their cellular effects [10] (Figure 1). The first three of the LPARs, LPAR1-3, belong to the Edg (endothelial differentiation gene) family and are ubiquitously expressed in most tissues [11]. LPAR4-6 belong to the P2Y puringenic receptor family and have not been as comprehensively studied [11]. LPA signaling is vital to cellular biology, as ATX knockout is embryonically lethal in murine models [12]. LPARs, however, have redundant and overlapping signaling pathways, as all murine knockouts for individual LPARs are viable [13,14]. As shown in Figure 1, this is supported by both the overlapping and compensatory signaling cascades that can be activated depending both on the G-proteins to which the LPARs are coupled and in which subsequent pathways LPA signaling is transduced. Double knockouts of all the LPAR1-3 receptors and a triple LPAR1-3 knockout are also all viable [15,16]. To date, only the double LPAR4/LPAR6 knockout is known to be embryonically lethal secondary to angiogenesis defects such as those observed in ATX knockout mouse fetuses [17].

In cancer biology, LPA signaling is a well-recognized mediator of cancer progression and loss of treatment efficacy, which is largely executed through the upregulation of multiple chronic inflammatory pathways [18]. Mechanistically, tumors upregulate LPA signaling through a combination of increased ATX production either by the cancer cells themselves or tumor stroma, autocrine overexpression of the LPAR receptors, or downregulation of total tumor lipid phosphate phosphatase (LPP) activity, which degrades LPA into monoacylglycerol [4]. The overall net effect of these actions is an increase in LPA concentrations and/or the receptor expression in the tumor microenvironment (TME) [7]. Consequently, there is a great deal of ongoing research into inhibiting the ATX–LPAR–LPP axis for the therapy of pancreatic cancer [19] and other chronic inflammatory diseases. This applies particularly to idiopathic pulmonary fibrosis, for which the ATX inhibitor GLPG1690 (ziritaxestast) and LPAR1 receptor antagonist BMS-986020 have entered clinical trials [20,21]. For in-depth details on the cancer and cell biology of LPARs and the history of inhibitor development against LPARs, we refer the reader to several excellent reviews [22,23,24,25]. We additionally refer the reader to the recent review by Geralo et al. for a detailed summary of LPAR expression and functional patterns and the molecular characteristics of the receptors [26].

Following numerous preclinical investigations in cell cultures and murine tumor models of different tumor sites conducted to elucidate the effects of LPAR signaling in cancer progression, broadly speaking, LPAR1 and LPAR4 are associated with cell motility and invasion, LPAR2 is associated with cell survival against cancer therapy, LPAR3 and LPAR6 are associated with tumor proliferation and antitumor immunity via the regulation of dendritic cell migration, and LPAR5 is associated with immune cell evasion [26]. However, there are no systematic studies on LPAR biology in human breast tumors. Deconvoluting the roles of the individual LPARs within the TME is critical prior to initiating clinical trials against a biological system wherein multiple receptors may have redundant signaling roles. An example of this can be seen in beta integrin signaling, wherein eight receptors exist [27]. Targeted monotherapy against the β1 integrin using the monoclonal antibody volociximab and other similar compounds have all failed clinical trials, likely secondary to complementary signaling by the remaining unimpeded beta integrins [28,29]. In this study, we explore the role of LPAR mRNA expression within the human breast cancer TME in three independent cohorts via in silico research approaches. These results can be used to make comparisons with preclinical investigations to aid ongoing pharmacological development efforts for the mitigation of deleterious LPAR signaling in cancers.

## 2. Results

### 2.1. Low LPAR1, LPAR4, and LPAR6 Gene Expression and High LPAR2 Gene Expression Correlate with a More Aggressive Breast Cancer Phenotype

**Figure 2:**
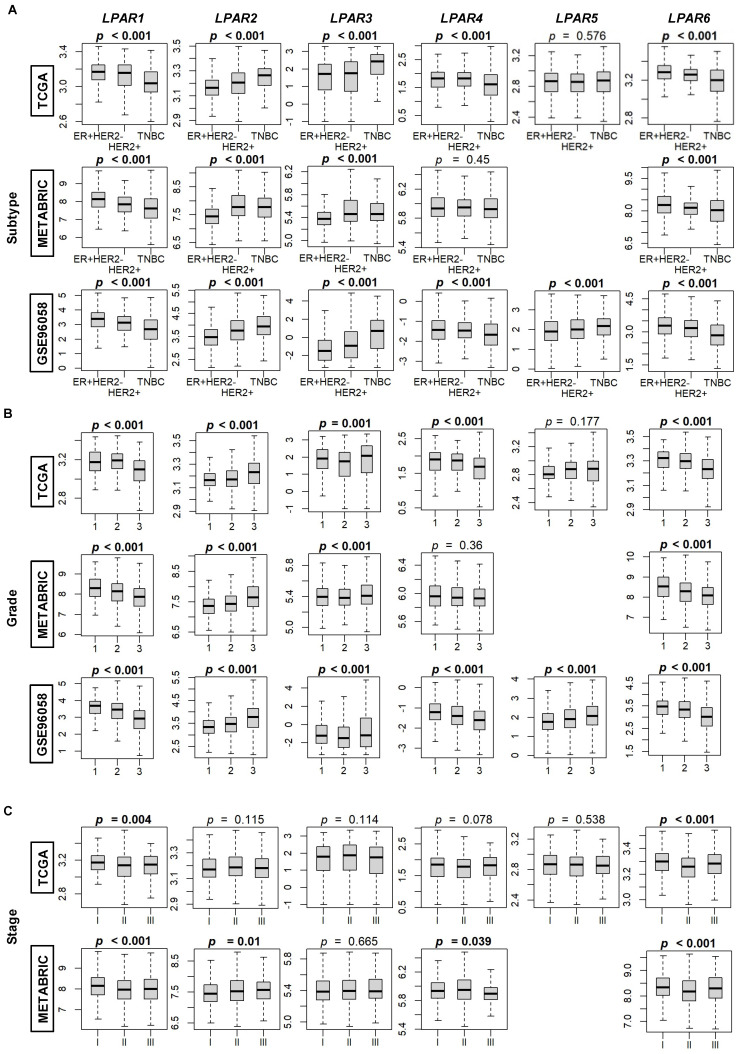
LPAR gene expression according to breast cancer subtype, grade, and stage. (**A**) Breast cancer subtypes: ER+HER2– (estrogen-receptor-positive, human-epidermal-growth-factor-receptor-negative), HER2+, and TNBC (triple-negative breast cancer). Counts segregated by cohort: TCGA (ER+HER2– *n* = 593, HER2+ *n* = 184, and TNBC *n* = 160), METABRIC (ER+HER2– *n* = 1355, HER2+ *n* = 236, and TNBC *n* = 313), and GSE96058 (ER+HER2– *n* = 2277, HER2+ *n* = 392, and TNBC *n* = 155). (**B**) Breast cancer grade—counts arranged by cohort: TCGA (Grade 1 *n* = 77, Grade 2 *n* = 269, and Grade 3 *n* = 235), METABRIC (Grade 1 *n* = 165, Grade 2 *n* = 740, and Grade 3 *n* = 927), and GSE96058 (Grade 1 *n* = 454, Grade 2 *n* = 1439, and Grade 3 *n* = 1115). (**C**) Breast cancer staging according to the American Joint Committee on Cancer (AJCC). Stage is not available for the GSE96058 cohort. Counts arranged by cohort: TCGA (Stage I *n* = 181, Stage II *n* = 617, and Stage III *n* = 248) and METABRIC (Stage I *n* = 475, Stage II *n* = 800, and Stage III *n* = 115). The bolded center bar within the box plots represents the median; the lower and upper box bounds represent the 25th and 75th percentiles, respectively; and the lower and upper tails represent the minimum and maximum values, respectively. *LPAR5* data are not available in the METABRIC cohort.

We began our analysis by correlating LPAR gene expression with breast cancer subtype. *LPAR1* and *LPAR6* gene expression were highest in ER+HER2– (estrogen-receptor-positive, human-epidermal-growth-factor-receptor-negative) tumors and lowest in TNBC (triple negative breast cancer) tumors in all three cohorts, while this pattern was reversed for *LPAR2* and *LPAR3* (all *p* < 0.001, Figure 2A). There was no consistent trend for *LPAR4* and *LPAR5* (Figure 2A). *LPAR1* and *LPAR6* gene expression was highest in grade 1 tumors and lowest in grade 3 tumors (all *p* < 0.001). Conversely, *LPAR2* and *LPAR3* gene expression was highest in grade 3 tumors across all three cohorts (all *p* < 0.001), with no trends for *LPAR4* or *LPAR5* (Figure 2B). According to stage, *LPAR1* and *LPAR6* gene expression was highest in stage 1 tumors and lowest in stage 3 tumors in both TCGA and METABRIC (GES96058 does not report stage data) (all *p* < 0.01, Figure 2C). There was no correlation between *LPAR2-5* expression and stage (Figure 2C). When examined with respect to proliferation, there was a statistically significant negative correlation between computational proliferation scores and Ki67 expression (an immunohistochemical marker of cell mitosis) for *LPAR1*, *LPAR4*, and *LPAR6* expression, whereas there was a significant positive correlation for *LPAR2*, and no correlation for *LPAR3* or *LPAR5* (all *p* < 0.001, Figure S1A,B). Finally, LPAR status did not correlate with node positivity, apart from a trend towards lower levels of *LPAR1* in node-positive tumors in the METABRIC and GSE96058 cohorts (both *p* < 0.05) but this was not significant in the TCGA cohort (Figure S1C). There were only 20 metastatic tumors in the TCGA cohort and 9 in the METABRIC cohort. Therefore, no conclusions can be drawn regarding the correlation between LPAR gene expression and metastatic disease (Figure S1D).

**Figure 3:**
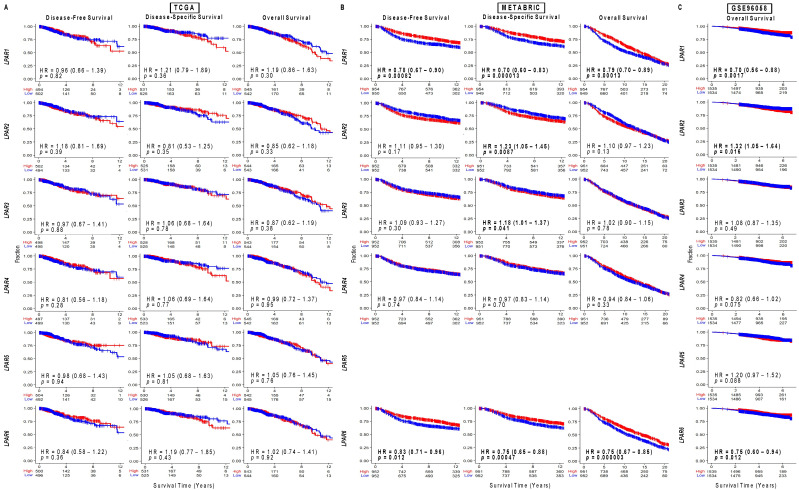
Survival plots for low and high LPAR gene expression in breast cancer tumors for the whole cohort for each dataset. (**A**) TCGA cohort results. (**B**) METABRIC cohort results. (**C**) GSE96058 cohort results. Patients at risk for each time point are listed along the *x*-axis. LPAR gene expression is dichotomized into low and high groups according to median values. The hazard ratio (HR) compares the high group against the low group. *LPAR5* data are not available in the METABRIC cohort.

We next examined survival trends between high and low LPAR receptor expression (dichotomized by median values). High *LPAR1* expression was correlated with improved disease-free survival (DFS), disease-specific survival (DSS), and overall survival (OS) in the METABRIC cohort (hazard ratios (HR)~0.7–0.8; 95% confidence intervals (CIs)~0.6–0.9) and increased OS in the GSE96058 cohort (HR 0.70 (0.56–0.88), Figure 3). However, significance was not reached in the TCGA cohort (Figure 3). The survival trends for *LPAR6* followed the same pattern, and the survival magnitude for all three cohorts was similar to that of *LPAR1* (Figure 3). For *LPAR2-5*, there were no consistently significant results across the three cohorts, but the survival trends favored increased survival among low-*LPAR2-* and low-*LPAR3-*expressing tumors (Figure 3). These results were then sub-analyzed by hormone status in Figures S2–S4, but there were no meaningful differences when compared to the overall cohort analysis in Figure 3.

**Figure 4:**
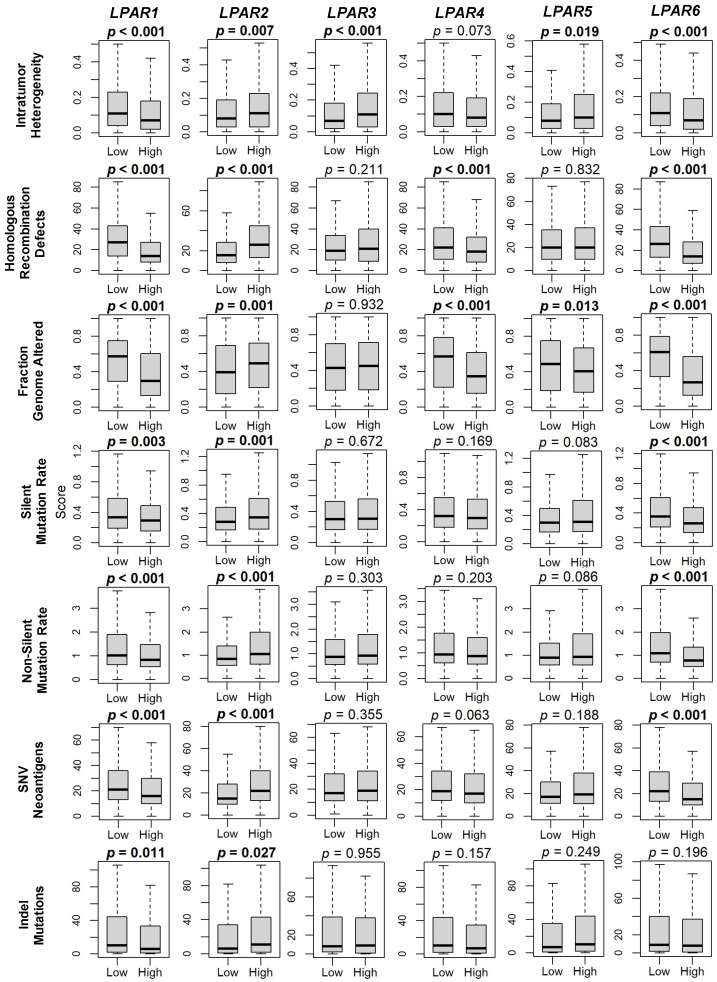
LPAR gene expression correlations with breast cancer mutations. Box plots of intratumor heterogeneity, homologous recombination defects, fraction-genome-altered, silent mutation rate, non-silent mutation rate, single-nucleotide variant (SNV) neoantigens, and indel mutations. Data derived from the scores obtained by Thorsson et al. [31]. LPAR gene expression is dichotomized into low and high groups according to the median. The bolded center bar represents the median; the lower and upper box bounds represent the 25th and 75th percentiles, respectively; and the lower and upper tails represent the minimum and maximum values, respectively. *LPAR5* data are not available in the METABRIC cohort.

Tumor mutational burden is a molecular surrogate that is commonly used to estimate cancer aggressiveness in many tumor types [30]. Therefore, we examined a panel of conventional tumor mutation makers (intratumor heterogeneity, homologous recombination defects (HRDs), fraction-genome-altered (FGR), silent mutation rate (SMR), non-silent mutation rate (NSMR), single-nucleotide variant (SNV) neoantigens, and indel mutations) and correlated them with LPAR gene expression. For *LPAR1*, all seven markers were significantly decreased in the high-expression group (all *p* < 0.02), and, similarly, in the high-*LPAR6* tumors (all *p* < 0.001), with the exception of indel mutations, which did not reach significance (*p* = 0.2, Figure 4). For *LPAR2*, the opposite trend occurred, where all seven markers were significantly increased in high-*LPAR2*-expressing tumors (all *p* < 0.01; Figure 4). There were no trends for either *LPAR3* or *LPAR5*, and all marker scores were decreased in *LPAR4*-high tumors but significantly so for only HRDs and FGR (both *p* < 0.001; Figure 4).

### 2.2. Low LPAR1, LPAR4, and LPAR6 Gene Expression and High LPAR2 Gene Expression Are Particularly Correlated with Increased Cell Cycle Signaling

**Figure 5:**
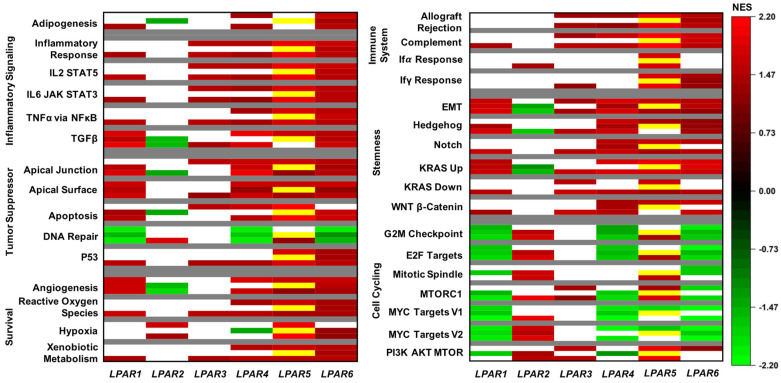
Gene set enrichment analysis (GSEA) for the LPARs in breast cancer. For all Hallmark gene sets listed, the top bar indicates the normalized enrichment score (NES) from the TCGA cohort, the middle bar indicates that from the METABRIC cohort, and the lower bar indicates that from the GSE96058 cohort. A false discovery rate (FDR) of less than 0.25 was considered statistically significant. White bars indicate no significance (FDR ≥ 0.25). Yellow bars indicate that *LPAR5* data are not available in the METABRIC cohort.

We performed gene set enrichment analysis (GSEA) on pathways within the Hallmark gene set [32] to determine LPAR gene expression. Gene sets were selected if they presented significant enrichment in at least two cohorts for any of the LPAR genes. The complete GSEA output is tabulated in Table S1. In general, *LPAR1*, *LPAR4*, and *LPAR6* presented a similar pattern of enriched cell cycle signaling in low-expressing tumors and enrichment in the DNA repair pathway in all three cohorts (Figure 5). Conversely, enriched cell-cycle-signaling pathways correlated with high *LPAR2* gene expression (Figure 5). Inflammatory signaling pathways were elevated, particularly in high-*LPAR6*-expressing tumors in all cohorts, and in tumor suppressor pathways apart from DNA repair pathways in high-*LPAR1-*, *LPAR4-*, and *LPAR6-*expressing tumors (Figure 5). Pattern correlations within survival, immune system, and stemness gene sets were more nuanced across the LPAR genes (Figure 5).

### 2.3. LPAR2 Is Predominantly Expressed in Cancers Cells, While the Other LPARs Are Expressed Primarily in the Stromal Cells in the Tumor Microenvironment

**Figure 6:**
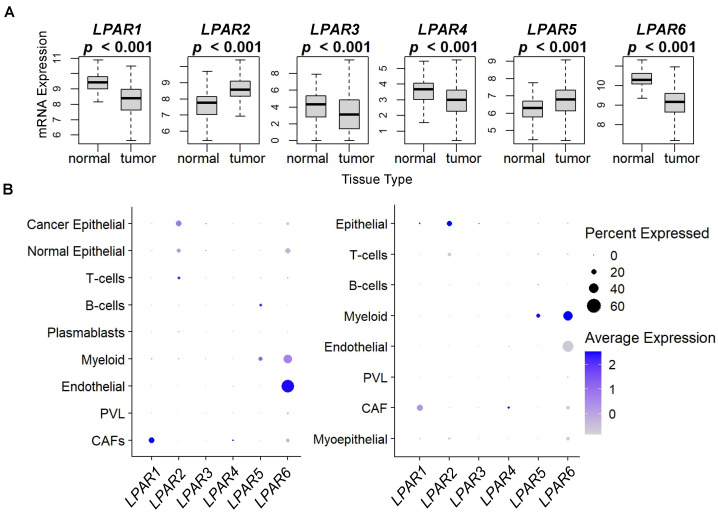
LPAR gene expression in normal tissues and tumors and dot plot analysis of LPAR expression for single-cell RNA sequencing of breast cancer tumors. (**A**) LPAR gene expression from 114 normal breast tissues is compared to 1090 breast cancer tumors from the TCGA database. The results are plotted as box plots. The bolded center bar represents the median; the lower and upper box bounds represent the 25th and 75th percentiles, respectively; and the lower and upper tails represent the minimum and maximum values, respectively. (**B**) Single-cell RNA-sequencing results from the cohort described in [33], comprising 26 tumors (11 ER+HER2−, 5 HER2+, and 10 TNBC), with a total of 130,246 single cells (left panel), and single-cell RNA sequencing results from the cohort described in [34], comprising 5 TNBC tumors, with a total of 24,271 single cells (right panel). For results in (**B**), the summary chart shows the overall percentage of the total LPAR expression arranged by cell type and the average expression within each cell type for each cohort.

Subsequently, we examined LPAR expression within the tumor microenvironment. First, we compared LPAR levels in normal breast tissue against breast tumors. The levels were significantly different between the two groups in all six LPAR genes (all *p* < 0.001), with the levels being lower in tumors compared to normal tissue for *LPAR1*, *LPAR3*, *LPAR4*, and *LPAR6* and higher in tumors compared to normal tissue for *LPAR2* and *LPAR5* (Figure 6A). We then further analyzed LPAR expression within the tumor microenvironment in two cohorts of single-cell RNA sequencing to determine which cells were the predominant expressors [33,34]. *LPAR1* and *LPAR4* expression levels were highest in cancer-associated fibroblasts (CAFs), while most of the *LPAR6* expression determined was in endothelial cells followed by myeloid cells and CAFs (Figure 6B and Figure S5). Regarding *LPAR2*, its highest expression was in cancer epithelial cells followed by normal epithelial cells, and *LPAR5* expression was highest in myeloid cells followed by B-cells (Figure 6B and Figure S5). *LPAR3* expression was not preferentially expressed in any cell type (Figure 6B and Figure S5).

**Figure 7:**
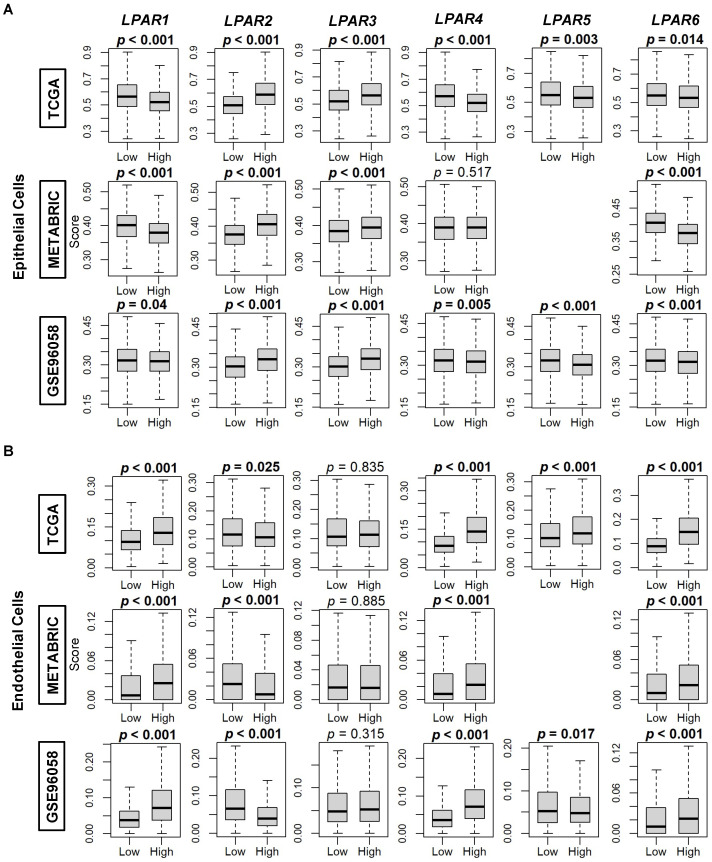
Epithelial cell and endothelial cell composition correlation with LPAR expression in breast cancer tumors. (**A**) Box plots of epithelial cell composition. (**B**) Box plots of endothelial cell composition. Box plots are based on the xCell algorithm for the TCGA, METABRIC, and GSE96058 cohorts. LPAR gene expression is dichotomized into low and high groups according to the median. The bolded center bar represents the median; the lower and upper box bounds represent the 25th and 75th percentiles, respectively; and the lower and upper tails represent the minimum and maximum values, respectively. *LPAR5* data are not available in the METABRIC cohort.

We further examined LPAR expression dichotomized by the median within tumor microenvironment cell populations using the xCell algorithm to perform cell type enrichment analysis. Epithelial cell (primarily cancer cells) composition was significantly enriched in high-*LPAR2* and -*LPAR3*-expressing tumors in all three cohorts (all *p* < 0.001) but decreased in high-*LPAR1*, -*LPAR5*, and -*LPAR6*-expressing tumors (all *p* < 0.05, Figure 7A). Similarly, epithelial cells were significantly enriched for high-*LPAR4*-expressing tumors in both the TCGA and GSE96058 cohorts, but this level did not reach significance in the METABRIC cohort (Figure 7A). When endothelial cells were analyzed, their composition was enriched in high-*LPAR1*, -*LPAR4*, and -*LPAR6-*expressing tumors and in low-expressing-*LPAR2* tumors in all three cohorts (all *p* < 0.05, Figure 7B). A virtually identical pattern of expression occurred in microvascular and lymphatic endothelial cells (Figure S6A,B). The same correlation pattern of enrichment in high-*LPAR1-*expressing tumors was also demonstrated for pericyte composition in all three cohorts (all *p* < 0.001) but not for the other LPARs (Figure S6C).

**Figure 8:**
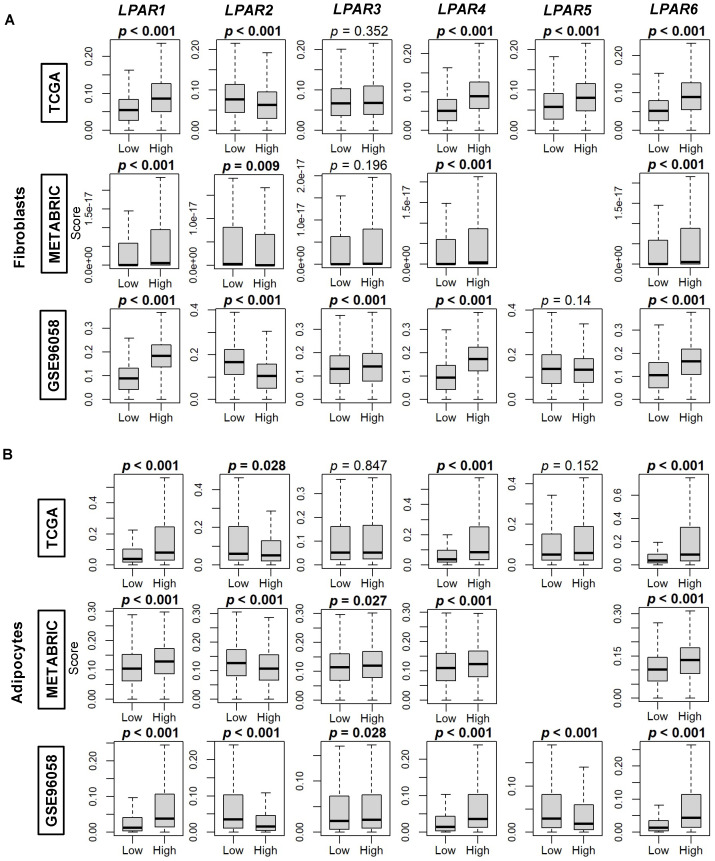
Fibroblast and adipocyte cell composition correlation with LPAR expression in breast cancer tumors. (**A**) Box plots of fibroblast composition. (**B**) Box plots of adipocyte composition. Box plots are based on the xCell algorithm for the TCGA, METABRIC, and GSE96058 cohorts. LPAR gene expression is dichotomized into low and high groups according to the median. The bolded center bar represents the median; the lower and upper box bounds represent the 25th and 75th percentiles, respectively; and the lower and upper tails represent the minimum and maximum values, respectively. *LPAR5* data are not available in the METABRIC cohort.

We then compared the correlation of LPAR gene expression with the xCell algorithm’s calculations of the composition of fibroblasts, adipocytes, and preadipocytes in breast cancer tumors. Fibroblast composition was significantly enriched in all three cohorts in high-*LPAR1*, -*LPAR4*, and -*LPAR6*-expressing tumors, while enrichment was correlated with low *LPAR2* levels (all *p* < 0.01, Figure 8A) Similarly, adipocytes were also enriched in high-*LPAR1-*, *LPAR4-*, and -*LPAR6*-expressing tumors and low-*LPAR2*-expressing tumors across all three cohorts (all *p* < 0.01, Figure 8B). Regarding preadipocytes, significant enrichment across the three cohorts only occurred in high-*LPAR6-*expressing tumors (all *p* < 0.001, Figure S7). TGF-β response, a marker of stromal fibrosis, was assessed via scores by Thorsson et al. [31]. The TGF-β response score was increased in the higher-expressing group of all the LPARs, except *LPAR2*, where tumors expressing high *LPAR2* had a lower TGF-β response (all *p* < 0.001, Figure S7). Similarly, the stromal fraction was significantly increased in the higher-expressing group of all the LPARs (all *p* < 0.001), except *LPAR2*, where there was no difference between the two groups (Figure S8).

### 2.4. LPAR5- and LPAR6-High Tumors Correlate with Increased Tumor Immune Cell Infiltration and Decreased Immune System Evasion

Lastly, we examined the correlation of immune cell populations with LPAR gene expression. For anti-cancer CD8+ cells, enrichment was only significantly correlated with high-*LPAR5-* and *LPAR6*-expressing tumors (all *p* < 0.001, Figure S9A). For T helper (Th)1 cells, the scores were significantly increased in low-*LPAR1-*, *LPAR4-*, *LPAR5-*, and *LPAR6-*expressing tumors and in high-*LPAR2*-expressing tumors across all cohorts (all *p* < 0.001, Figure S9B). M1 macrophage expression was significantly decreased in high-*LPAR1-* and *LPAR4-*expressing tumors and increased in *LPAR2-* and *LPAR5*-expressing tumors (all *p* < 0.001, Figure S9C). For dendritic cells, scores were increased in all highly expressing tumors across all LPARs (all *p* < 0.05) except for *LPAR2*, where there was no significance across the three cohorts (Figure S9D). Pro-tumor T regulatory cells (Tregs) did not show any consistent trends among the LPARs (Figure S10A). For Th2 cells, these were enriched in low-*LPAR1-* and *LPAR4-*expressing tumors and in high-*LPAR2*-expressing tumors in all three cohorts (all *p* < 0.001, Figure S10B), and for M2 macrophages, they were enriched in low-*LPAR2-* and *LPAR3*-expressing tumors and in high-*LPAR5*-expressing tumors (all *p* < 0.05, Figure S10C).

**Figure 9:**
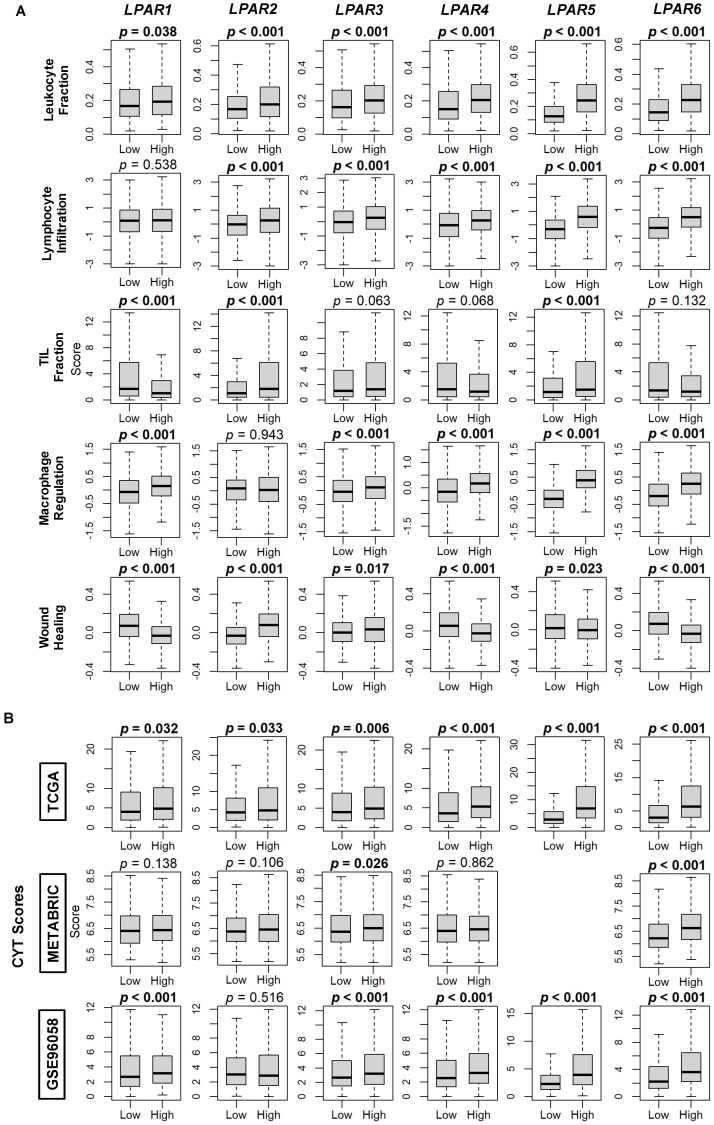
Immune scores for markers of tumor immune cell populations and cytolytic (CYT) score correlations with LPAR gene expression in breast cancer tumors. (**A**) Box plots of immune score results are based on scores reported by Thorsson et al. [31]. (**B**) Box plots of CYT scores are based on the results obtained using the xCell algorithm applied to the TCGA, METABRIC, and GSE96058 cohorts. *LPAR5* data are not available in the METABRIC cohort. LPAR gene expression is dichotomized into low and high groups according to the median. The bolded center bar represents the median; the lower and upper box bounds represent the 25th and 75th percentiles, respectively; and the lower and upper tails represent the minimum and maximum values, respectively.

Overall, when examining the immune-related scores reported by Thorsson et al. [31], the enrichment of the tumor leukocyte fraction was significantly correlated with the high expression group for all the LPARs (all *p* < 0.001), and the same also occurred for the lymphocyte infiltration score (all *p* < 0.001), except for *LPAR1*, for which there was no difference between the low and high groups (Figure 9A). TIL fraction was decreased in high-*LPAR1-*expressing tumors and increased in high-*LPAR2-* and *LPAR5-*expressing tumors, while macrophage scores were increased in high expressing tumors for all the LPARs except *LPAR2* (all *p* < 0.001, Figure 9A). Wound-healing scores were decreased in high- *LPAR1-*, *LPAR4-*, *LPAR5-*, and *LPAR6-*expressing tumors and increased in high-*LPAR2* and *LPAR3*-expressing tumors (all *p* < 0.02, Figure 9A). Finally, upon analysis of cytolytic (CYT) activity, high-*LPAR5-* and *LPAR6-*expressing tumors had significantly increased CYT scores across the cohorts (all *p* < 0.05, Figure 9B).

## 3. Discussion

The majority of cancer research in the LPA field has focused on its production by the enzyme ATX and the subsequent phenotypical effects mediated by global LPA signaling [6,35]. These effects essentially amplify cancer progression through the upregulation of numerous pro-inflammatory and pro-survival pathways that ultimately fuel metastatic potential and treatment therapy [36]. Therefore, most of the pharmaceutical research on inhibiting LPA signaling has focused on ATX inhibitor development for both cancer and inflammatory conditions such as pulmonary idiopathic fibrosis [37]. The ATX inhibitor, IOA-289, is currently in phase 1b clinical trials for pancreatic cancer and is the first inhibitor of the ATX–LPA–LPP axis to be specifically tested in cancer patients [19]. However, efforts are ongoing to produce compounds that can target this axis at multiple levels [38,39]. A combination therapy of both a potent and long-lasting ATX inhibitor along with an appropriately selective LPAR blockade, potentially incorporated as a single compound as in the case of ongoing efforts to design dual ATX-LPAR1 inhibitors [39], should provide a robust adjunct therapy that can mitigate cancer treatment failure or side effects, such as radiation-induced fibrosis in breast cancer adjuvant radiotherapy [40]. To date, the LPAR1 inhibitors BMS-986020 and BMS-986278 have entered clinical trials for idiopathic pulmonary fibrosis [21,41], while the LPAR1 inhibitor SAR100842 has been included in clinical trials for reducing skin sclerosis in patients with diffuse cutaneous systemic sclerosis [42]. It remains to be seen if LPAR inhibitors, either alone or in combination with ATX inhibitors, might have useful adjunct effects in improving cancer therapies.

In this study, we have examined LPAR expression within the breast TME in three large independent cohorts incorporating over 5000 patients, representing the largest and most detailed study to date. The key findings are summarized in Table 1. *LPAR1*, the most studied LPAR, had the lowest expression in TNBC, grade 3 tumors, and stage III disease, and high*-LPAR1*-expressing tumors had a lower overall mutational burden. Taken together, patients with high expression *LPAR1* tumors had a significant survival advantage compared to patients with low expression *LPAR1* tumors, with an HR of 0.70–0.79 in the METABRIC and GSE96058 cohorts but not in the TCGA cohort. This is likely due to the overall enrichment of ER+HER2– cancers in the METABRIC and GSE96058 cohorts. High-*LPAR4-* and *LPAR6-*expressing tumors also had a similar phenotype to *LPAR1*. A major commonality among these receptors is that they are more highly expressed in normal breast tissue and tumor stroma than the cancer cells themselves (Table 1). Among the other LPARs, *LPAR2* demonstrated the most robust expression in tumors compared to normal breast tissue and was the only LPAR to be expressed primarily in cancer epithelial cells (Table 1). *LPAR2* expression was highest in TNBCs and grade 3 tumors, and high-expressing-*LPAR2* tumors had universally higher mutational burden rates (Table 1) with increased cycle cycling gene set enrichment, particularly with respect to G2M, E2F, mitotic spindle, and myc targets. Taken together, these results suggest that LPAR2 signaling may be the most specific among the LPARs in mediating pro-cancer pathological processes.

These findings regarding LPAR2 are supported in cell culture and murine models of breast cancer. In cell culture assays, TNBC growth was most dependent on the autocrine-produced inflammatory cytokines IL6, IL8, and CXCL1 in an NFkappaB-dependent manner. This signaling could be entirely blocked by LPAR2 inhibition alone [43]. In a seminal murine tumor study examining the overexpression of ATX and LPAR1, LPAR2, and LPAR3 in a mouse mammary tumor virus model, the LPAR2-overexpressing model had the highest tumorgenicity rate at 52.8%, followed by ATX at 50.0%, LPAR3 at 42.3%, and LPAR1 at 32.0% [44]. Taken together, these findings suggest that LPAR2 inhibition may have a unique specificity for mitigating breast cancer tumor progression. Such an inhibitor, especially when combined with anti-ATX treatment, might offer selective therapeutic benefits in breast cancer patients.

This study has several limitations. Despite using three large, independent cohorts to validate our results, our analysis is retrospective and comprised of heterogenous patient populations presenting with various outcomes. While these results from real patient data can provide invaluable insights to complement pre-clinical investigations, we cannot necessarily imply mechanisms of action with bioinformatic data. Ultimately, the delineation of the influences of individual LPARs on TME biology will require the development and systematic testing of receptor-specific inhibitors in representative tumor models. It is also unknown how LPARs might change either in expression level or function with disease progression, metastasis, and/or the development of treatment resistance. Virtually all the tumors within the analyzed cohorts of this study are early or locoregional treatment-naive breast cancers. Analysis of temporal and therapy-induced effects on breast tumor biology would require serial measurements of LPAR mRNA either from tumor tissue or circulating tumor-free transcriptome analysis.

Regardless, the results from this investigation provide a robust and systematic baseline for delineating complementary and opposing factors with respect to LPAR signaling to aid in the rational design of potential pharmaceutical adjuncts. Since redundancy in cell signaling usually has evolutionary significance with respect to function, deeper research into the role of LPAR signaling in TME biology is warranted. Such investigations may lead to the development of novel adjunct therapies for treating cancer and other chronic inflammatory-mediated conditions.

## 4. Materials and Methods

### 4.1. Data Acquisition

Clinical outcomes and mRNA expression for breast cancer patients were obtained from three large databases: the Cancer Genome Atlas Program (TCGA) (whole database *n* = 1090; estrogen-receptor-positive and human-epidermal-growth-factor-receptor-negative (ER+ HER2–) *n* = 593; HER2+ *n* = 184; triple-negative breast cancer (TNBC) *n* = 160), the Molecular Taxonomy of Breast Cancer International Consortium (METABRIC) (whole database *n* = 1094, ER+ HER2– *n* = 1355, HER2+ *n* = 236, and TNBC *n* = 313), and GSE96058 (whole database *n* = 3069, ER+ HER2– *n* = 2277, HER2+ *n* = 392, and TNBC *n* = 155). These data were retrieved from the cBioPortal (https://www.cbioportal.org (accessed on 22 September 2022)) and the Gene Expression Omnibus (GEO) repository of the United States National Institutes of Health (https://www.ncbi.nlm.nih.gov/geo (accessed on 22 September 2022)), as previously described [45,46]. Gene expression data from 114 normal breast tissue samples were retrieved from the Genotype-Tissue Expression (GTex) Portal (https://gtexportal.org (accessed on 22 September 2022)) [47]. Single-cell RNA-sequencing breast cancer atlas data were sourced [33,34] via the Broad Institute Single-Cell Portal (https://singlecell.broadinstitute.org/single_cell (accessed on 22 September 2022)). As all data were sourced from deidentified public resources, the Roswell Park Institutional Review Board waived ethics approval.

### 4.2. Gene Set Enrichment Analysis

Functional enrichment analysis of LPAR genes (*LPAR1-6*) was conducted via gene set enrichment analysis (GSEA) [33] applied to the Molecular Signatures Database Hallmark collection (http://www.gsea-msigdb.org (accessed on 22 September 2022)) [32]. A false discovery rate (FDR) < 0.25 specified gene sets with enriched signaling [48]. High- and low-LPAR-expression groups were dichotomized by median gene expression. Positive NES scores indicate enriched signaling in the high-LPAR-expression group, and negative NES scores indicate enriched signaling in the low-LPAR-expression group.

### 4.3. Other Scores

The xCell algorithm (https://xcell.ucsf.edu (accessed on 22 September 2022)) [49] correlated LPAR gene expression with the TME stromal cell infiltration fractions, as previously described [50,51,52,53,54]. Stromal cell populations examined included adipocytes, preadipocytes, fibroblasts, endothelial cells, and pericytes) and immune cells (CD8+, T helper cell (Th)1 and Th2 cells, T-regulator cells, M1 and M2 macrophages, and dendritic cells). Markers of tumor mutation (intratumor heterogeneity, homologous recombination defects, fraction-genome-altered, silent mutation rate, non-silent mutation rate, single-nucleotide neoantigens, and indel mutations), proliferation score, stromal fraction, TGF-β score, and immune scores (leukocyte fraction, lymphocyte infiltration, tumor infiltration lymphocyte fraction, macrophage regulation, and wound healing) were obtained from Thorsson et al. [31]. TME Immune cytolytic activity (CYT) was calculated as the geometric mean of the expression of perforin (*PRF1*) and granzyme A (*GZMA*) mRNA expression, which measures the anti-cancer ability of cytotoxic T cells [55].

### 4.4. Statistical Analyses

Statistical analyses were conducted using R 4.2.1 (https://www.R-project.org (accessed on 22 September 2022)). Graphics were produced with R 4.2.1 and Origin Pro 2022 (OriginLab Corporation, Northampton, MA, USA). LPAR gene expression was dichotomized into low and high groups based on the median. All results are plotted as box plots, with the lower and upper bounds representing the maximum and minimum values, the upper and lower ends of the box representing the 25th and 75th percentile values, and the bolded bar within the box representing the median value. Two-group comparisons were performed using the Wilcoxon signed-rank test, and multiple-group comparisons were conducted using the Kruskal–Wallis test. R 4.2.1 was used to analyze disease-free survival (DFS), disease-specific survival (DSS), and overall survival (OS) based on high or low LPP expression via Cox-proportional hazards regression. *p* < 0.05 was set for statistical significance.

## Figures and Tables

**Table 1:** Summary table of primary findings of this study.

	*LPAR1*	*LPAR2*	*LPAR3*	*LPAR4*	*LPAR5*	*LPAR6*
Most Common Subtype	ER+HER2–	TNBC	TNBC	–	–	ER+HER2–
Most Common Grade	Grade 1	Grade 3	Grade 3	Grade 1	–	Grade 1
Highest Proliferation (Low vs. High LPAR Group)	Low	High	–	Low	–	Low
Highest Mutation Burden (Low vs. High LPAR Group)	Low	High	–	–	–	Low
Normal vs. Tumor (Highest Expression)	Normal	Tumor	Normal	Normal	Tumor	Normal
TME Cell Type (Highest Expression)	CAFs	Cancer Epithelial	–	CAFs	Myeloid	Endothelial
Highest Cytolytic Activity (Low vs. High LPAR Group)	–	–	High	–	High	High

## Data Availability

All datasets are publicly available via cBioPortal (https://www.cbioportal.org (accessed on 22 September 2022)) or the Gene Expression Omnibus (GEO) repository of the United States National Institutes of Health (https://www.ncbi.nlm.nih.gov/geo (accessed on 22 September 2022)), and single cell cohort data via the Broad Institute Single Cell Portal (https://singlecell.broadinstitute.org/single_cell (accessed on 22 September 2022)).

## Supplementary Materials

The supporting information can be downloaded at: https://www.mdpi.com/article/10.3390/ijms24129812/s1.
