# Supplementary material for: Lysophosphatidic Acid Receptor Signaling in the Human Breast Cancer Tumor Microenvironment Elicits Receptor-Dependent Effects on Tumor Progression
Source: Int J Mol Sci. 2023 Jun 6;24(12):9812. doi: 10.3390/ijms24129812 (PMC10298074; doi:10.3390/ijms24129812)
Supplement: Supplementary file 1 [file ijms-24-09812-s001.zip › LRAR Supplemental Paper submission.pdf]

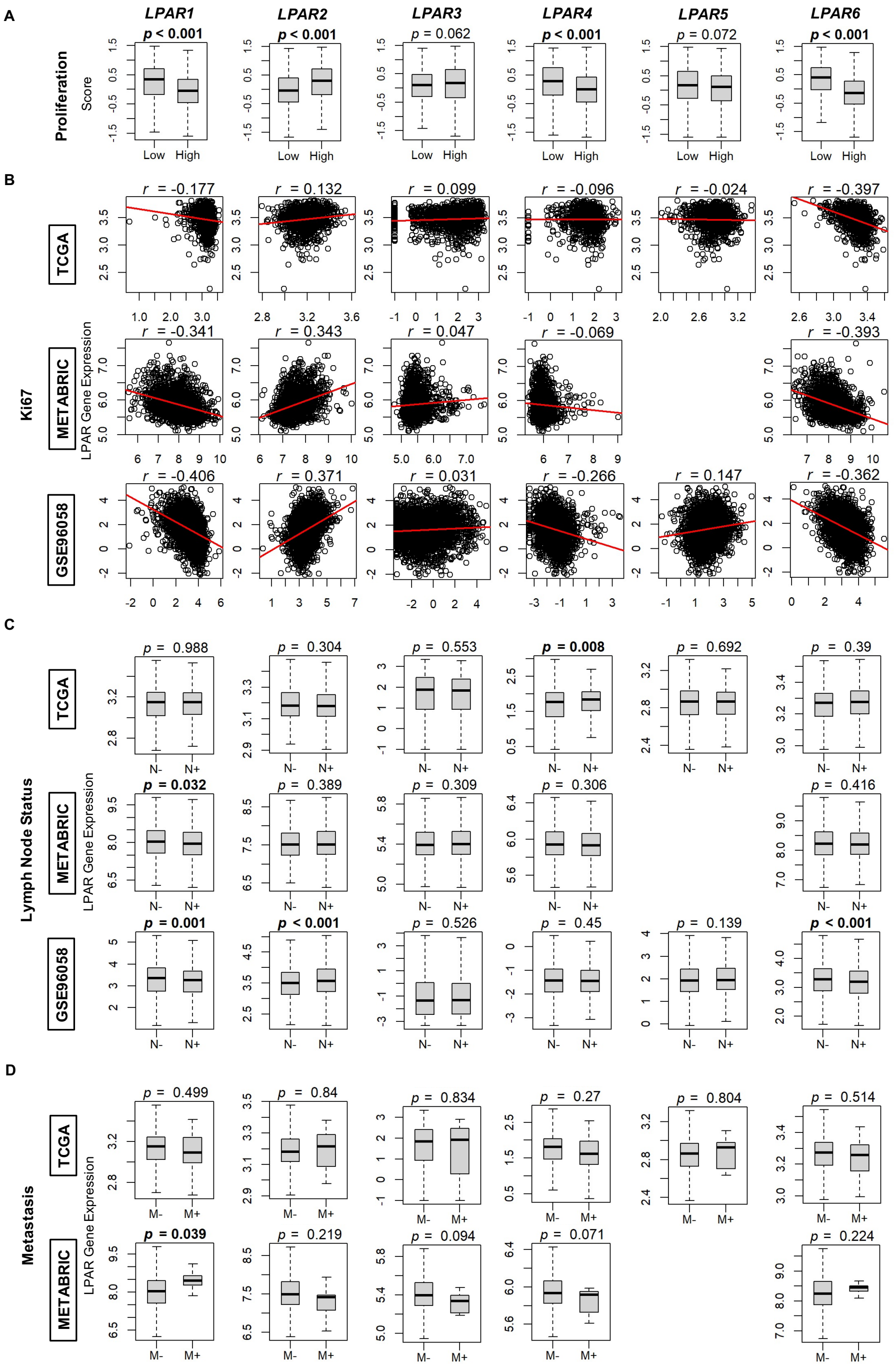

Figure S1. LPAR gene expression by breast cancer tumor proliferation, nodal status, and metastasis. (**A**) Proliferation score. Derived from the scores by Thorsson, et al. [1]. (**B**) Ki67 correlation plots to LPAR expression. (**C**) Tumor nodal status (negative or positive). Counts by cohort: TCGA (N– (negative) *n* = 513, N+ (positive) *n* = 557), METABRIC (N– *n* = 993, N+ n = 911), GSE96058 (N– *n* = 1811, N+ *n* = 1162). (**D**) Tumor metastasis (negative or positive). Counts by cohort: TCGA (M– (negative) *n* = 1046, M+ (positive) *n* = 20), METABRIC (M– *n* = 1394, M+ *n* = 9). Because stage is not available for the GSE96058 cohort, metastasis data is unavailable for this cohort. The bolded center bar within the box plots represents the median; the lower and upper box bounds represent the 25th and 75th percentiles, respectively; and the lower and upper tails represent the minimum and maximum values, respectively. *LPAR5* data is not available in the METABRIC cohort.

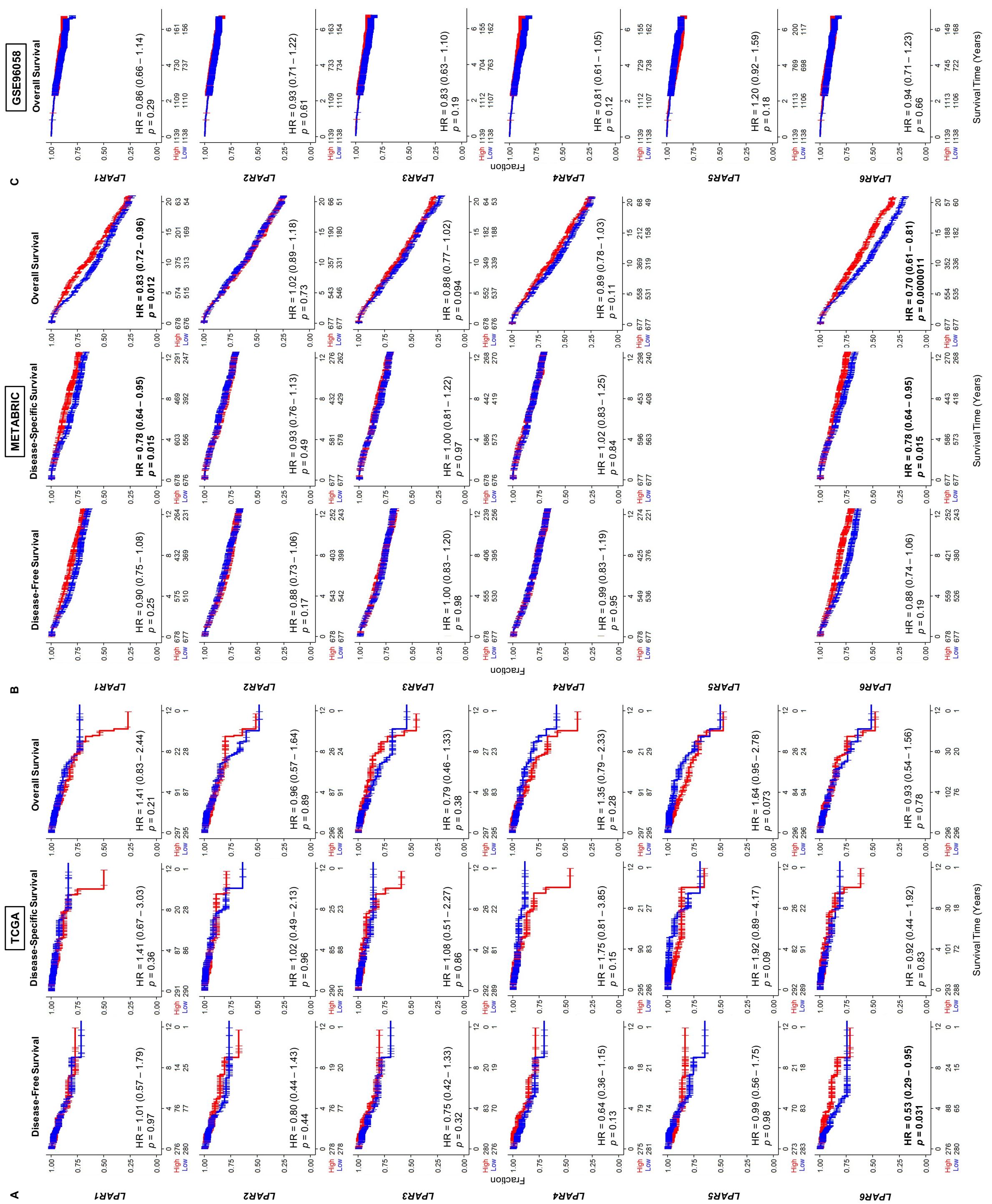

Figure S2. Survival plots for low and high LPAR gene expression in breast tumors for the estrogen receptor (ER) positive, human epidermal growth factor receptor (HER2) negative cohort for each dataset. (**A**) TCGA cohort results. (**B**) METABRIC cohort results. (**C**) GSE96058 cohort results. Patients at risk for each time point are listed along the x-axis. LPAR expression is dichotomized into low and high groups by the median. The hazard ratio (HR) compares the high group against the low group. *LPAR5* data is not available in the METABRIC cohort.

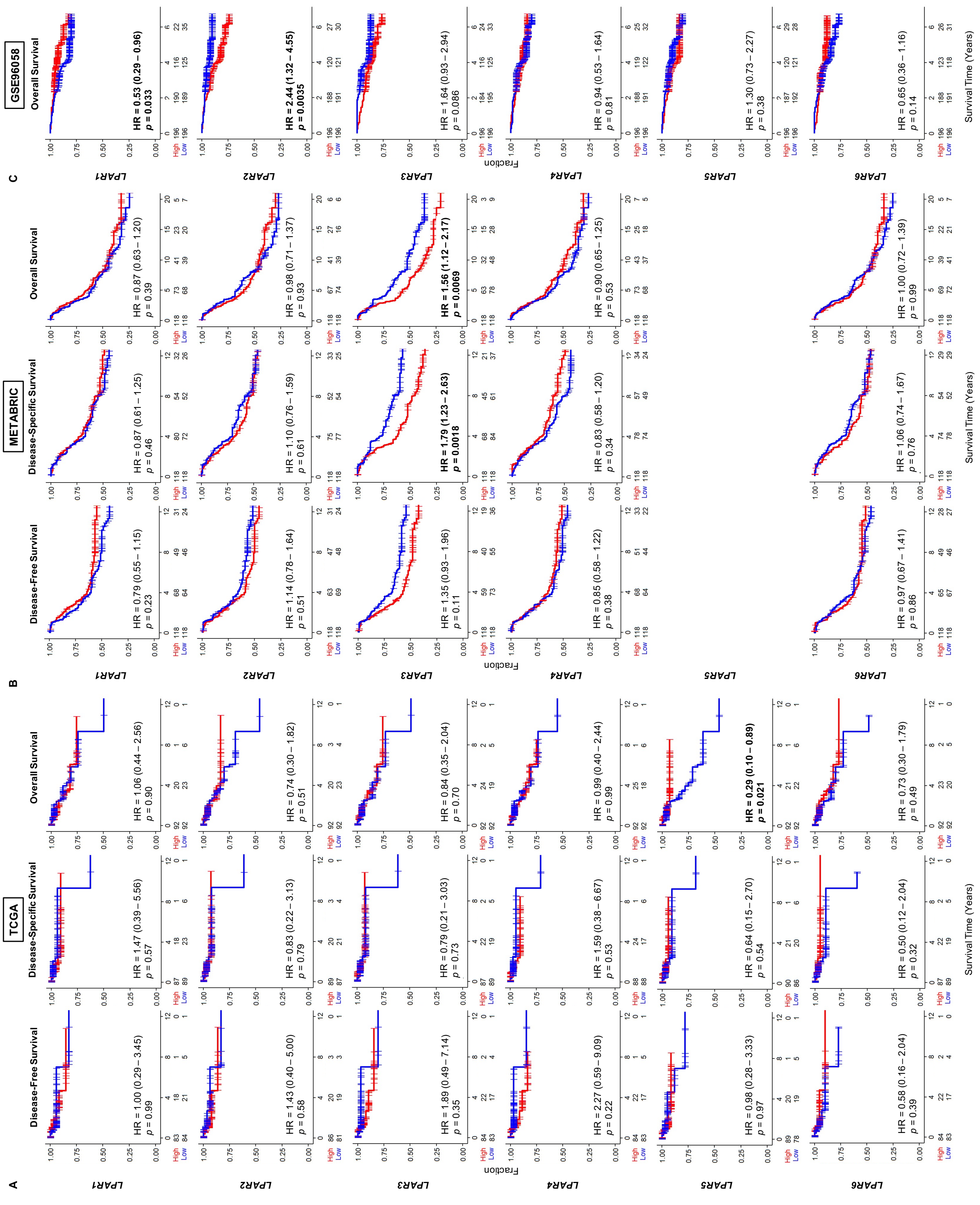

Figure S3. Survival plots for low and high LPAR gene expression in breast tumors for the human epidermal growth factor receptor (HER2) positive cohort for each dataset. (**A**) TCGA cohort results. (**B**) METABRIC cohort results. (**C**) GSE96058 cohort results. Patients at risk for each time point are listed along the x-axis. LPAR expression is dichotomized into low and high groups by the median. The hazard ratio (HR) compares the high group against the low group. *LPAR5* data is not available in the METABRIC cohort.

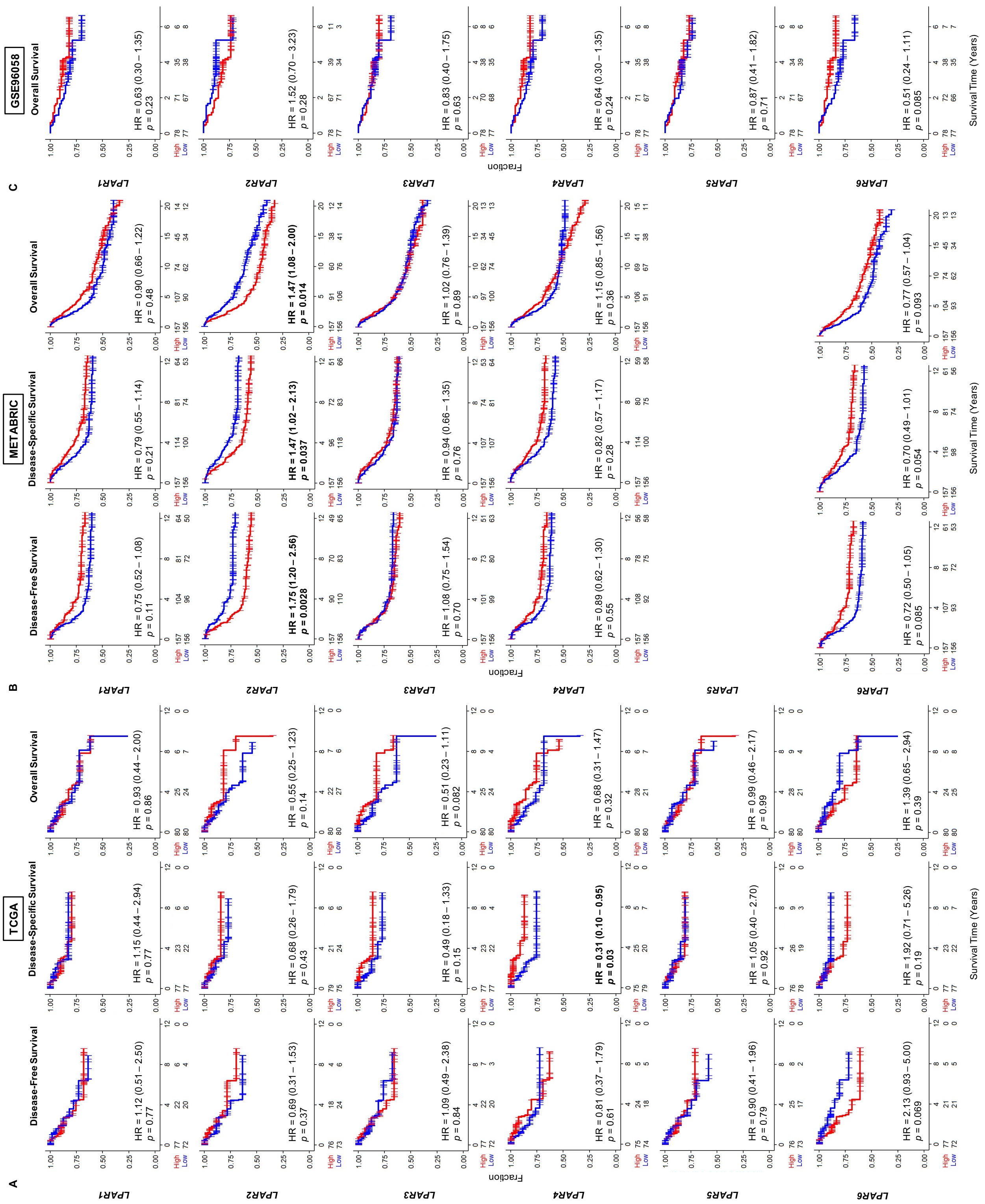

Figure S4. Survival plots for low and high LPAR gene expression in breast tumors for the triple negative breast cancer (TNBC) cohort for each dataset. (**A**) TCGA cohort results. (**B**) METABRIC cohort results. (**C**) GSE96058 cohort results. Patients at risk for each time point are listed along the x-axis. LPAR expression is dichotomized into low and high groups by the median. The hazard ratio (HR) compares the high group against the low group. *LPAR5* data is not available in the METABRIC cohort.

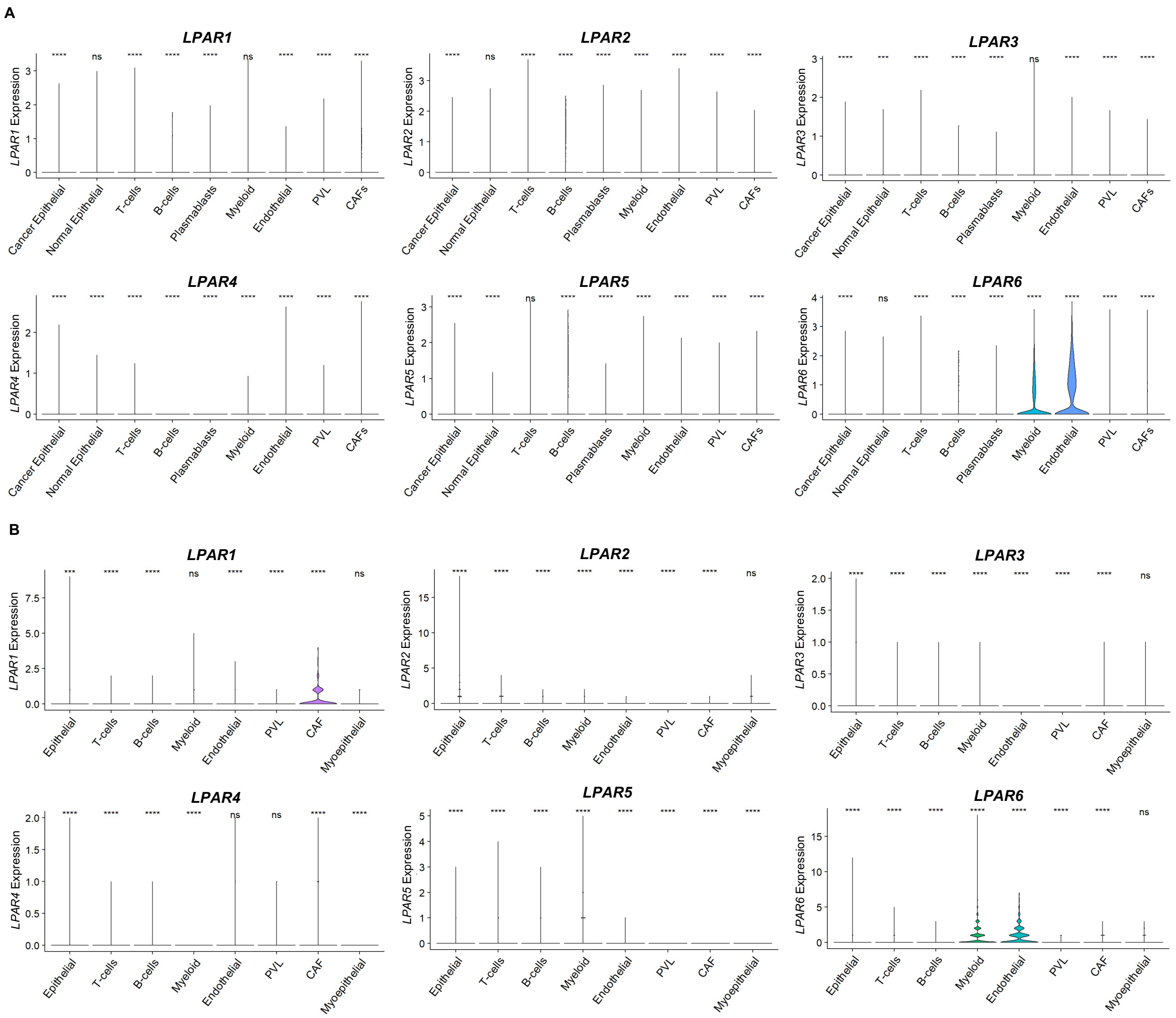

Figure S5. Violin plot analysis of LPAR gene expression via singe cell RNA sequencing of breast cancer tumors. (**A**) Single-cell RNA sequencing results from the cohort described in [2], comprised of 26 tumors (11 ER+ HER2−, 5 HER2+, and 10 TNBC), with a total of 130,246 single cells. (**B**) Single-cell RNA sequencing results from the cohort described in [3], comprised of 5 TNBC tumors, with a total of 24,271 single cells. Results for the individual LPAR genes are shown following *t*-test for each type compared with the base mean (**** $p \leq 0.0001$; ns, not significant).

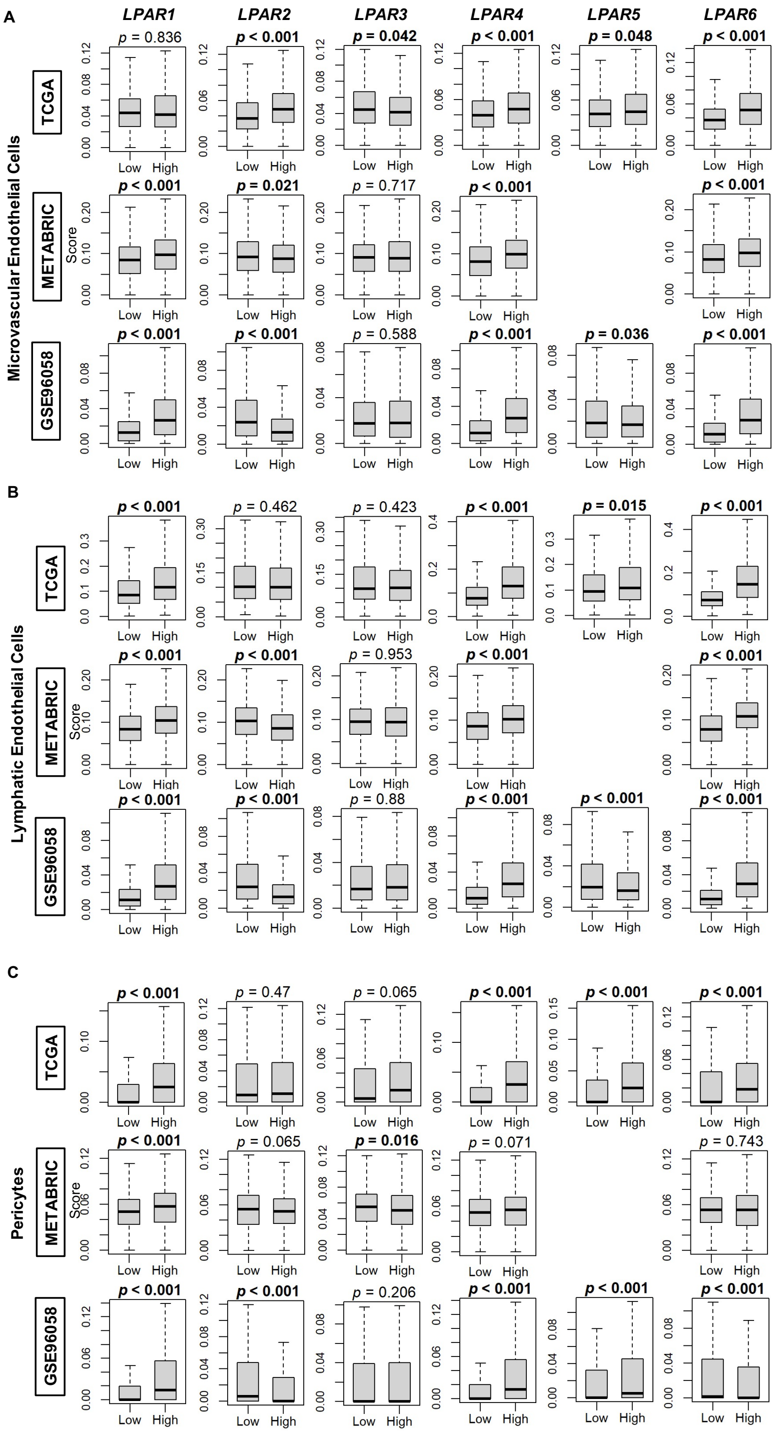

Figure S6. Microvascular endothelial cell, lymphatic endothelial cell, and pericyte composition correlation with LPAR gene expression in breast cancer tumors. (**A**) Box plots of microvascular endothelial cell composition. (**B**) Box plots of lymphatic endothelial cell composition. (**C**) Box plots of pericyte composition. All data are based on the xCell algorithm for the TCGA, METABRIC, and GSE96058 cohorts. LPAR gene expression is dichotomized into low and high groups by the median. The bolded center bar represents the median; the lower and upper box bounds represent the 25th and 75th percentiles, respectively; and the lower and upper tails represent the minimum and maximum values, respectively. *LPAR5* data is not available in the METABRIC cohort.

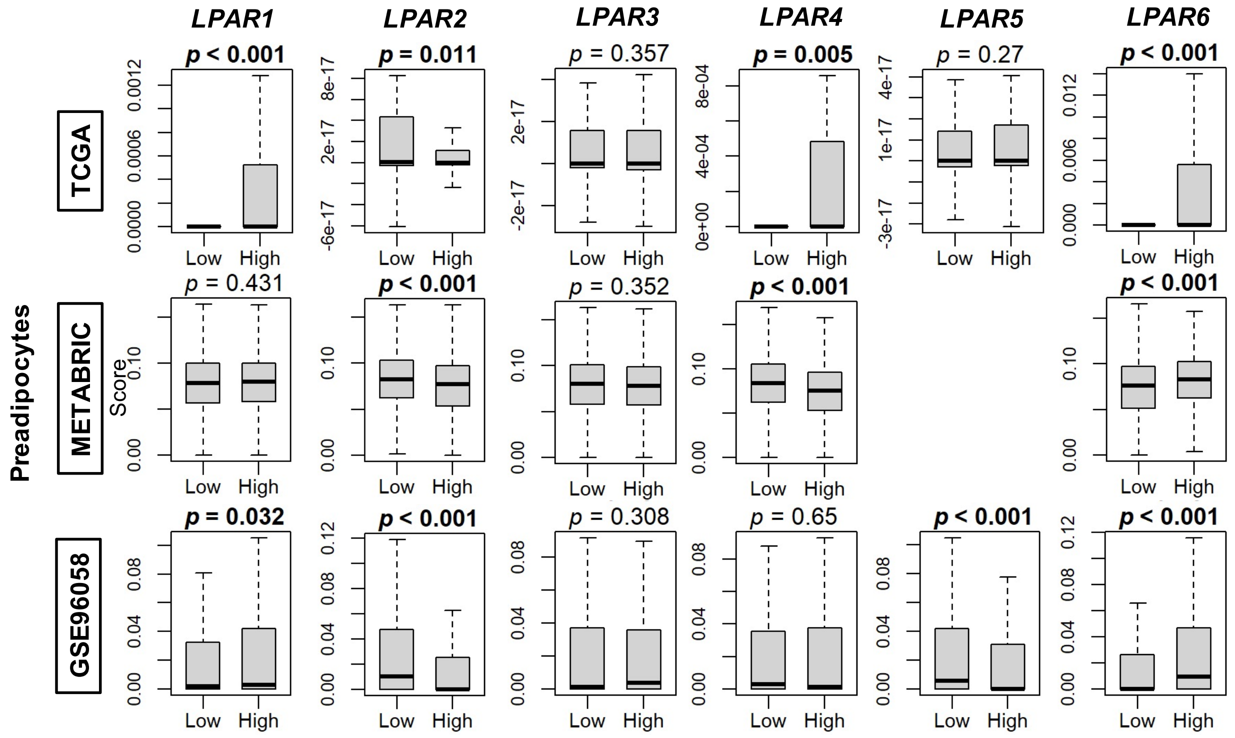

Figure S7. Preadipocyte composition correlation with LPAR gene expression in breast cancer tumors. Results are derived from the xCell algorithm for the TCGA, METABRIC, and GSE96058 cohorts. LPAR gene expression is dichotomized into low and high groups by the median. The bolded center bar represents the median; the lower and upper box bounds represent the 25th and 75th percentiles, respectively; and the lower and upper tails represent the minimum and maximum values, respectively. *LPAR5* data is not available in the METABRIC cohort.

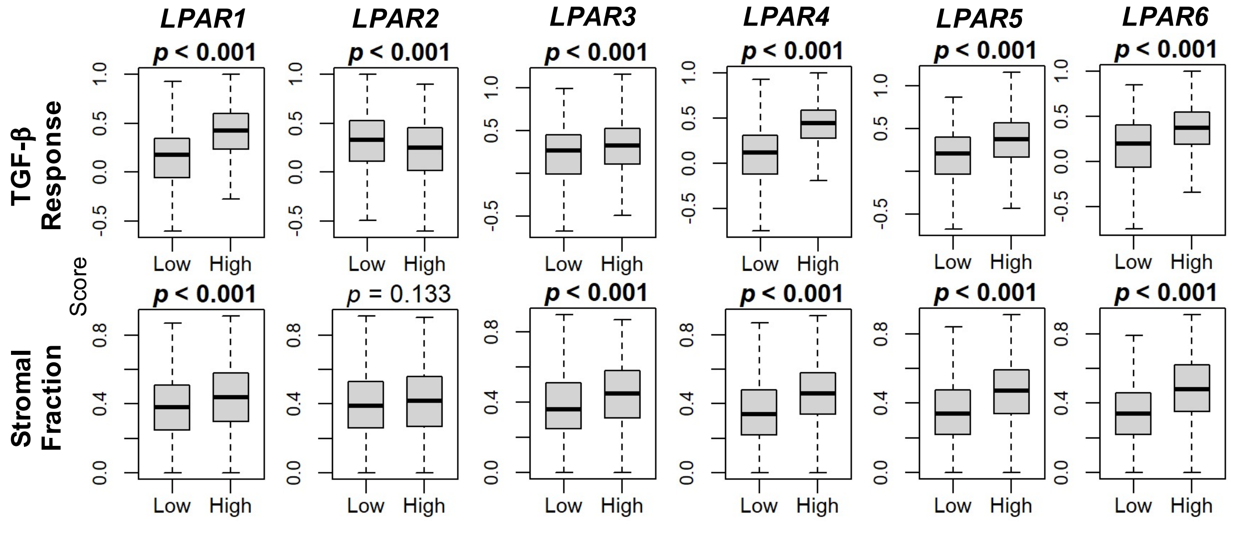

Figure S8. TGF-β and stromal fraction scores correlation with LPAR gene expression in breast cancer tumors. Derived from the scores by Thorsson, et al. [1]. LPAR gene expression is dichotomized into low and high groups by the median. The bolded center bar represents the median; the lower and upper box bounds represent the 25th and 75th percentiles, respectively; and the lower and upper tails represent the minimum and maximum values, respectively.

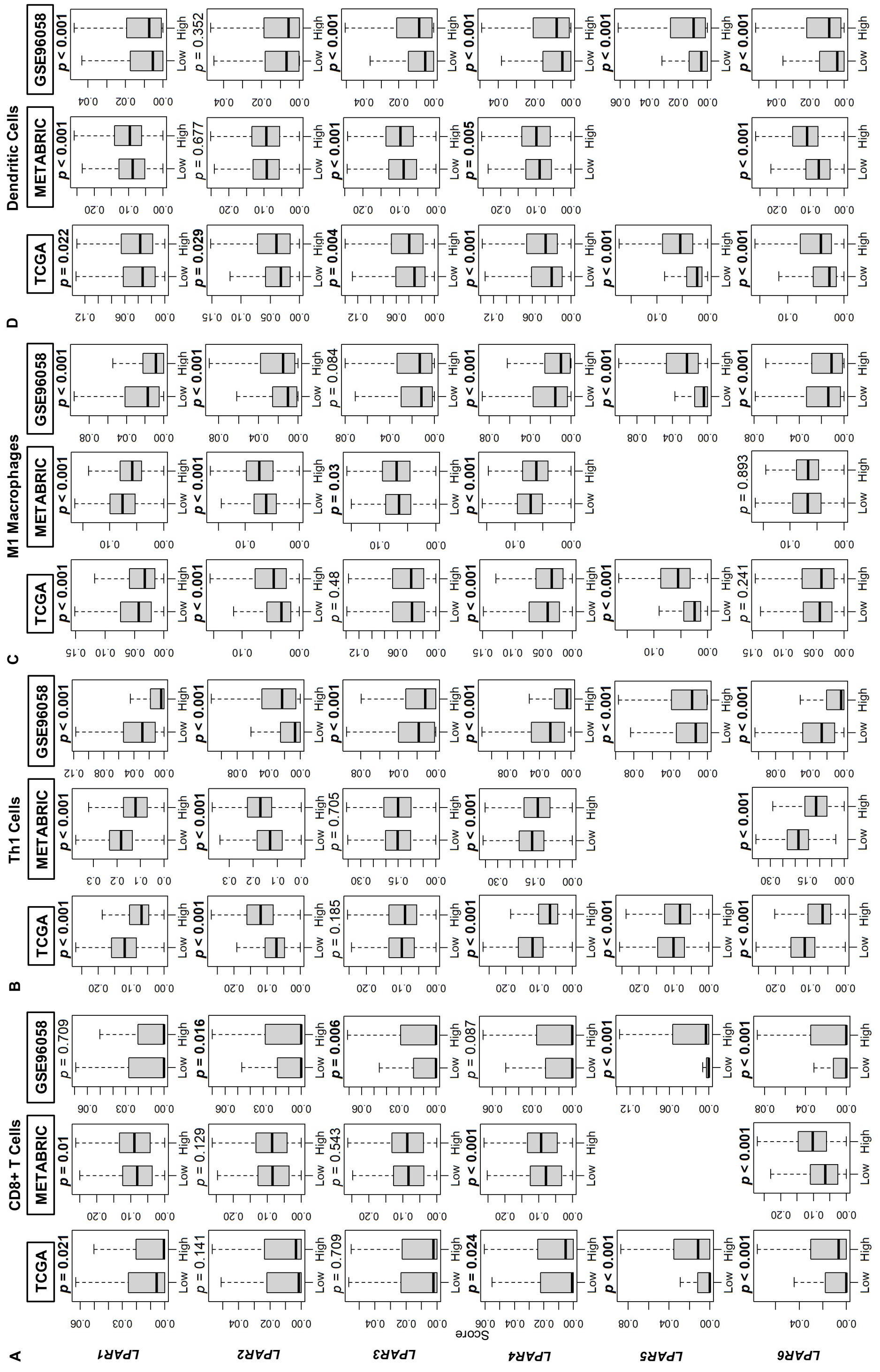

Figure S9. Anti-cancerous immune cell correlation with LPAR gene expression in breast cancer tumors. (**A**) Box plots of CD8+ T cell composition. (**B**) Box plots of T-helper 1 (Th1) cell composition. (**C**) Box plots of M1 macrophage composition. (**D**) Box plots of dendritic cell composition. All data based on the xCell algorithm for the TCGA, METABRIC, and GSE96058 cohorts. LPAR gene expression is dichotomized into low and high groups by the median. The bolded center bar represents the median; the lower and upper box bounds represent the 25th and 75th percentiles, respectively; and the lower and upper tails represent the minimum and maximum values, respectively. *LPAR5* data is not available in the METABRIC cohort.

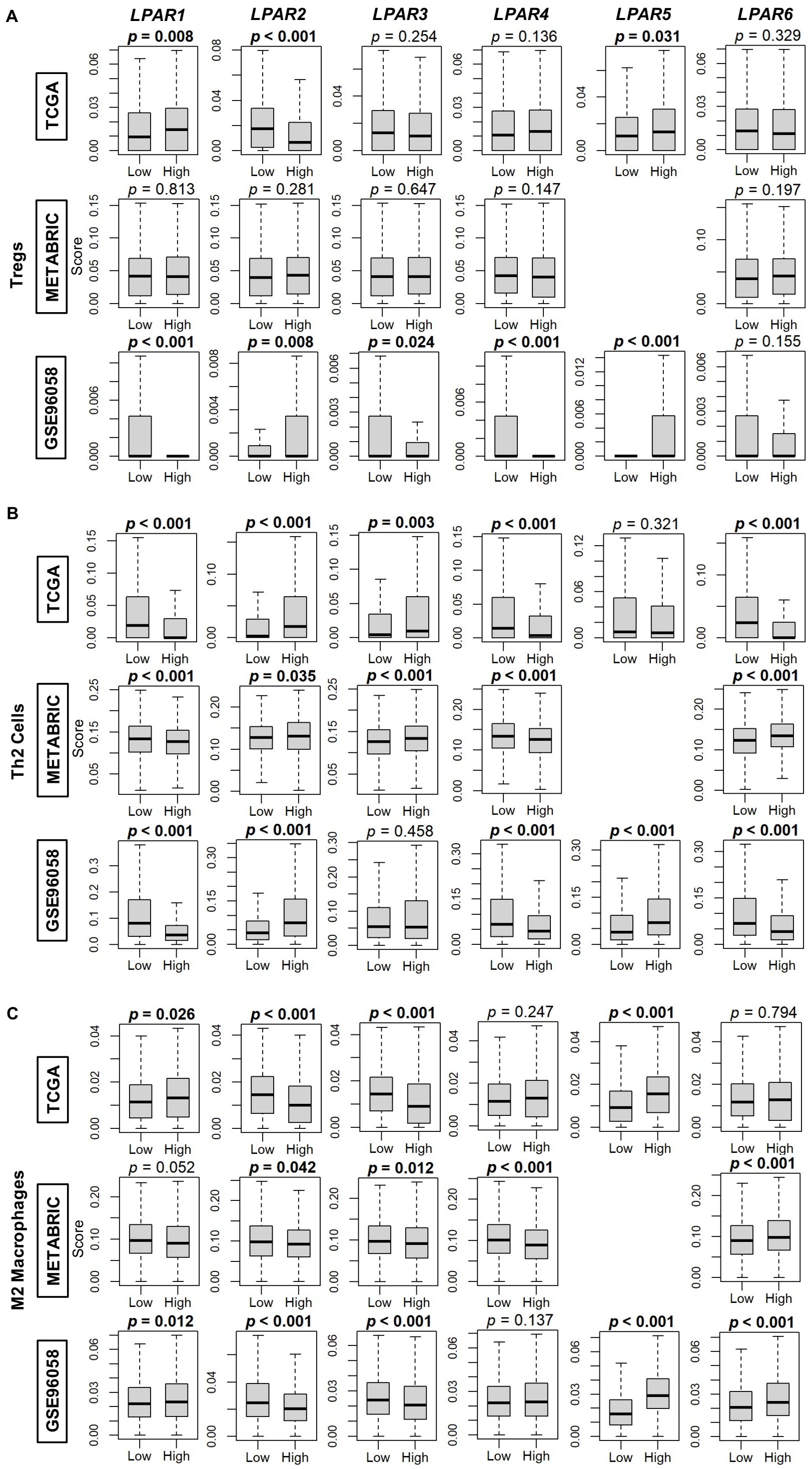

Figure S10. Pro-cancerous immune cell correlation with LPAR gene expression in breast cancer tumors. (**A**) Box plots of T regulatory (Treg) cell composition. (**B**) Box plots of T-helper 2 (Th2) cell composition. (**C**) Box plots of M2 macrophage composition. All data based on the xCell algorithm for the TCGA, METABRIC, and GSE96058 cohorts. LPAR gene expression is dichotomized into low and high groups by the median. The bolded center bar represents the median; the lower and upper box bounds represent the 25th and 75th percentiles, respectively; and the lower and upper tails represent the minimum and maximum values, respectively. *LPAR5* data is not available in the METABRIC cohort.

References

1. Thorsson, V.; Gibbs, D.L.; Brown, S.D.; Wolf, D.; Bortone, D.S.; Ou Yang, T.H.; Porta-Pardo, E.; Gao, G.F.; Plaisier, C.L.; Eddy, J.A.*, et al.* The immune landscape of cancer. *Immunity* **2018**, *48*, 812-830.e814.
2. Wu, S.Z.; Al-Eryani, G.; Roden, D.L.; Junankar, S.; Harvey, K.; Andersson, A.; Thennavan, A.; Wang, C.; Torpy, J.R.; Bartonicek, N.*, et al.* A single-cell and spatially resolved atlas of human breast cancers. *Nat. Genet.* **2021**, *53*, 1334-1347.
3. Wu, S.Z.; Roden, D.L.; Wang, C.; Holliday, H.; Harvey, K.; Cazet, A.S.; Murphy, K.J.; Pereira, B.; Al-Eryani, G.; Bartonicek, N.*, et al.* Stromal cell diversity associated with immune evasion in human triple-negative breast cancer. *EMBO J.* **2020**, *39*, e104063.
